# Associations between maternal lipid profile and pregnancy complications and perinatal outcomes: a population-based study from China

**DOI:** 10.1186/s12884-016-0852-9

**Published:** 2016-03-21

**Authors:** Wen-Yuan Jin, Sheng-Liang Lin, Ruo-Lin Hou, Xiao-Yang Chen, Ting Han, Yan Jin, Li Tang, Zhi-Wei Zhu, Zheng-Yan Zhao

**Affiliations:** Department of Children’s Health Care, Children’s Hospital, Zhejiang University School of Medicine, Hangzhou, 310003 China; Department of Epidemiology and Biostatistics, School of Public Health, Curtin University, Perth, WA6845 Australia

## Abstract

**Background:**

Dyslipidemia in pregnancy are associated with gestational diabetes mellitus (GDM), preeclampsia, preterm birth and other adverse outcomes, which has been extensively studied in western countries. However, similar studies have rarely been conducted in Asian countries. Our study was aimed at investigating the associations between maternal dyslipidemia and adverse pregnancy outcomes among Chinese population.

**Methods:**

Data were derived from 934 pairs of non-diabetic mothers and neonates between 2010 and 2011. Serum blood samples were assayed for fasting total cholesterol (TC), triglycerides (TG), high-density lipoprotein-cholesterol (HDL-C), and low-density lipoprotein-cholesterol (LDL-C) concentrations during the first, second and third trimesters. The present study explored the associations between maternal lipid profile and pregnancy complications and perinatal outcomes. The pregnancy complications included GDM, preeclampsia and intrahepatic cholestasis of pregnancy (ICP); the perinatal outcomes included preterm birth, small/large for gestational age (SGA/LGA) infants and macrosomia. Odds ratios (ORs) and 95 % confidence intervals (95 % CIs) were calculated and adjusted via stepwise logistic regression analysis. Optimal cut-off points were determined by ROC curve analysis.

**Results:**

After adjustments for confounders, every unit elevation in third-trimester TG concentration was associated with increased risk for GDM (OR = 1.37, 95 % CI: 1.18-1.58), preeclampsia (OR = 1.50, 95 % CI: 1.16-1.93), ICP (OR = 1.28, 95 % CI: 1.09-1.51), LGA (OR = 1.13, 95 % CI: 1.02-1.26), macrosomia (OR = 1.19, 95 % CI: 1.02-1.39) and decreased risk for SGA (OR = 0.63, 95 % CI: 0.40-0.99); every unit increase in HDL-C concentration was associated with decreased risk for GDM and macrosomia, especially during the second trimester (GDM: OR = 0.10, 95 % CI: 0.03-0.31; macrosomia: OR = 0.25, 95 % CI: 0.09-0.73). The optimal cut-off points for third-trimester TG predicting GDM, preeclampsia, ICP, LGA and SGA were separately ≥3.871, 3.528, 3.177, 3.534 and ≤2.530 mmol/L. The optimal cut-off points for third-trimester HDL-C identifying GDM, macrosomia and SGA were respectively ≤1.712, 1.817 and ≥2.238 mmol/L.

**Conclusions:**

Among Chinese population, maternal high TG in late pregnancy was independently associated with increased risk of GDM, preeclampsia, ICP, LGA, macrosomia and decreased risk of SGA. Relative low maternal HDL-C during pregnancy was significantly associated with increased risk of GDM and macrosomia; whereas relative high HDL-C was a protective factor for both of them.

## Background

Evidence has suggested that adverse pregnancy outcomes can jeopardize short- and long-term maternal and fetal health. Women with previous gestational diabetes mellitus (GDM) have an increased risk of type 2 diabetes or cardiovascular diseases (CVD) in later life [1, 2]. Pregnancies accompanied with preeclampsia have the predisposition to future CVD and metabolic syndrome [3–5]. According to Developmental origins of Health and Disease (DOHaD) hypothesis, individuals who were exposed to an undernourished intrauterine environment are more prone to developing type 2 diabetes or CVD in later life as a result of epigenetic modifications, which permanently reprogramme their bodies [6, 7]. Therefore, it is of great importance to explore effective interventions to prevent these adverse outcomes. It is noteworthy that disturbed maternal metabolism, including atherogenic lipid changes in pregnancy, is one of the crucial factors that involved in pathological process.

From the 12^th^ week of pregnancy, lipid parameters, including total cholesterol (TC), triglycerides (TG), low-density lipoprotein-cholesterol (LDL-C), high-density lipoprotein-cholesterol (HDL-C) and phospholipid gradually increase, especially in the second and third trimesters [8–13]. Previous research has indicated that pregnancy-induced hyperlipidemia contributes to an increased morbidity of GDM and preeclampsia. At all gestational stages, high TG concentrations were associated with a raised risk for gestational impaired glucose tolerance (GIGT) and GDM [14–16]. Results of a few studies have suggested a concentration-dependent positive association between maternal TG and the risk for preeclampsia [17–20]. Moreover, evidence has shown pregnant women with higher oxidized LDL-C concentrations are also at greater risk of developing preeclampsia [21–23]. In addition, Amsterdam Born Children and Their Development cohort study discovered that maternal TG concentrations in early pregnancy were linearly related with the prevalence of pregnancy-induced hypertension, preeclampsia, induced preterm birth (PTB) and large for gestational age (LGA) [24].

Except for pregnancy complications, maternal dyslipidemia is also closely linked with adverse perinatal outcomes. Catov et al. [25] reported that both elevated cholesterol and TG in early pregnancy were associated with an increased risk of spontaneous PTB. Furthermore, maternal impaired TG and non-esterified fatty acids (NEFA) metabolism were correlated with excessive fetal growth [26, 27]. In women diagnosed with GDM, it has been demonstrated that maternal TG and NEFA concentrations in late pregnancy are positively correlated with newborns’ birth weight, body mass index (BMI) and fat mass [28–30]. Interestingly, other clinical trials have indicated maternal fasting TG is positively correlated with neonatal body weight even in women with normal glucose tolerance but positive diabetic screenings [31, 32]. Additionally, recent studies have described a positive association between maternal TG concentrations and the risk for LGA newborns independent of glycemic control [24, 31, 33].

Despite these findings, there are still some controversies on the relationship between maternal lipid disturbances and certain pregnancy complications or perinatal outcomes. For instance, GDM was discovered to be associated with lower maternal LDL-C and HDL-C concentrations during the second and third trimesters in several studies [15, 34–36]. Nevertheless, other assays showed no significant difference in the concentrations of LDL-C and HDL-C between GDM and normal pregnancies [34, 36–38]. Additionally, as mentioned before, there exists a debate focused on whether maternal TG is positively correlated with neonatal birth weight only in GDM/diabetic subjects or also in nondiabetic ones [28–33, 39]. These conflicting results could potentially be explained by differences in trimester of pregnancy, condition of glycemic control, race/ethnicity and sample size, but the real causes remain unknown.

Our study was aimed at comprehensively investigating the associations between Chinese maternal lipid profile and adverse pregnancy outcomes, i.e. GDM, preeclampsia, intrahepatic cholestasis of pregnancy (ICP), PTB, LGA, small for gestational age (SGA) and macrosomia. At the same time, we attempted to advance understandings and provide some potent evidence for elucidating the existing controversies.

## Methods

### Study population

Between 30 June 2010 and 30 June 2011, pregnant women who attended regular prenatal health care and intended to give a birth in Women’s Hospital, Zhejiang University School of Medicine were invited to participate in the study. Before enrollment, our study was approved by the hospital’s Clinical Research Ethics Committee and written informed consent was signed by every participant. We established the study cohort based on inclusion and exclusion criteria. Inclusion criteria of pregnant women were: 1) pregnant at 28–37 gestational weeks; 2) had integrated medical records and clear gestational age; 3) singleton pregnancy; and 4) naturally conceived. Exclusion criteria of pregnant women were: 1) multiple pregnancy; 2) had diabetes mellitus, chromosomal abnormalities, inherited metabolic diseases or thyroid diseases before pregnancy; 3) experienced serious infection during early pregnancy; and 4) conceived with assisted reproductive techniques. A total of 1020 expectant mothers were recruited. All the women included were requested to complete an extensive questionnaire about maternal age, height, parity, prepregnancy weight, gestational weight gain (GWG), lifestyle, education background, family income and other important information. Data on first-trimester and second-trimester lipid concentrations, GDM, preeclampsia and ICP were collected from medical records. They were followed from recruitment to delivery. Information on delivery mode, gestational age, newborn sex, birth weight, Apgar scores and perinatal outcomes were recorded by midwives or obstetricians upon delivery. Inclusion criteria for newborns were singleton and 5-min-postpartum Apgar scores ≥ 7. Exclusion criteria for newborns were chromosomal abnormalities, inherited metabolic diseases and congenital abnormalities. Finally, 934 pairs of mothers and neonates were eligible for our study.

### Biochemical analyses

Venous blood samples were taken after overnight fasting from all the participants at the first (7–10 gestational weeks), second (21–24 gestational weeks) and third (33–37 gestational weeks) trimester of pregnancy. The blood samples were collected in a 3.5-ml vacutainer (BD Plymouth, PL6 7BP, UK, SST II Advance Tubes) for the preparation of serum. At the biochemical laboratory of Women’s Hospital, Zhejiang University School of Medicine, 1-ml aliquots of serum samples were obtained by centrifugation (3500 rpm for 10 min at 4 °C) and stored at −20 °C until analysis. Every sample was assayed for TC, TG, HDL-C and LDL-C concentrations. TC was assayed with the cholesterol oxidase-phenol aminophenazone method and TG was assayed using the glycerol-3-phosphatase oxidase-phenol aminophenazone method. HDL-C and LDL-C were measured by homogeneous enzymatic colorimetric assays. All the lipid measurements were performed on an automatic biochemical analyser (Abbott Architect C16000, Abbott Laboratories, USA) respectively with TC, TG, HDL-C and LDL-C detection kits (Abbott Diagnostic Kit, Abbott Laboratories, USA). The inter- and intra-assay coefficients of variation were <1.9 %, 0.81 % (TC); <1.9 %, 1.6 % (TG); <1.0 %, 0.44 % (HDL-C) and <1.9 %, 1.5 % (LDL-C), respectively.

### Definitions

BMI was calculated by dividing weight in kilograms by the square of height in meters. Maternal prepregnancy BMI was categorized into underweight (<18.5 kg/m^2^), normal weight (18.5-24.9 kg/m^2^), overweight (25.0-29.9 kg/m^2^), and obese (≥30.0 kg/m^2^) groups on the basis of World Health Organization BMI classification [40]. According to the new recommendations from American Institute of Medicine, GWG was stratified into appropriate, inadequate and excessive groups [41]. Based on different prepregnancy BMIs, appropriate GWG was defined as 12.5-18.0 kg in underweight women, 11.5-16.0 kg in normal weight women, 7.0-11.5 kg in overweight women and 5.0-9.0 kg in obese women. Exceeding the thresholds was defined as excessive GWG, while falling below the thresholds was defined as inadequate GWG.

GDM was diagnosed according to the recommendations from International Association of Diabetes and Pregnancy Study Groups [42, 43]. The proposed criteria for GDM were as follows: Before an oral glucose tolerance test (OGTT), every participant was requested for a 50 g glucose challenge test and serum glucose levels were assayed 1 hr later. Subjects with positive results (glucose levels ≥7.8 mmol/L) were required to undergo a 75 g OGTT. Serum glucose levels during OGTT were measured at 0, 1 and 2 hr, respectively. The normal values were fasting glucose <5.1 mmol/L, 1-hr glucose <10.0 mmol/L and 2-hr glucose <8.5 mmol/L. If one or more values equaled or exceeded the above thresholds, women were diagnosed as having GDM.

Hypertensive disorders in pregnancy were a set of diseases including pregnancy-induced hypertension, preeclampsia, eclampsia, chronic hypertension complicating pregnancy and preeclampsia superimposed upon chronic hypertension. Preeclampsia was defined as a specific pregnancy-induced disorder characterized with hypertension (systolic blood pressure ≥140 mmHg and/or diastolic blood pressure ≥90 mmHg) and significant proteinuria (urine protein ≥300 mg/24 h or positive results in random urine protein tests) in previously normotensive women on or after 20 weeks gestational age [44].

ICP is a pregnancy-specific disorder typically occurs in the third trimester characterized by pruritus and jaundice. Confirmation of diagnosis relied on abnormal liver function tests and raised maternal serum bile acids [45]. Abnormal liver function tests included elevated levels of alanine aminotransferase, aspartate aminotransferase and/or gamma-glutamyl transpeptidase. The upper limits of total serum bile acids were 10–14 micromoles/L in postprandial state and 6–10 micromoles/L in fasting state. Exceeding the upper limits was an important diagnostic basis of ICP.

Preterm birth was defined as birth of newborns less than 37 weeks gestational age. Newborns were classified into appropriate for gestational age (AGA), SGA and LGA on the basis of Neonatal Birth Weight for Gestational Age and Percentile in 15 Cities of China [46]. Neonates were defined as SGA if their birth weights fell below the 10^th^ percentile for gestational age and as LGA if their birth weights exceeded the 90^th^ percentile for gestational age. AGA was defined as birth weights met or exceeded the 10^th^ percentile and met or fell below the 90^th^ percentile for gestational age. According to the birth weight, neonates could be stratified into low birth weight (<2500 g), normal birth weight (2500–4000 g) and macrosomia (>4000 g) groups.

### Statistical analysis

In our study, normally and non-normally distributed continuous variables were respectively presented as mean ± standard deviation (SD) and median (interquartile range, IQR), categorical variables were presented as N (%). Serum TC, TG, LDL-C, HDL-C concentrations at the first, second and third trimesters were compared by Kruskal-Wallis test. Maternal lipid increases from the first to second trimester and the second to third trimester were compared using Mann–Whitney test. Forward stepwise logistic regression analysis were applied to explore the associations between maternal dyslipidemia and pregnancy complications and perinatal outcomes. We considered GDM, preeclampsia and ICP as pregnancy complications; and PTB, SGA, LGA, macrosomia as perinatal outcomes. In the multivariable adjusted model, maternal age, prepregnancy BMI, GWG, parity, cigarette exposure, socioeconomic status, infant sex and delivery mode were regarded as confounding variables. ROC curve analysis was conducted to determine the optimal cut-off points of TG, HDL-C and LDL-C for predicting maternal and neonatal adverse outcomes. Each optimal cut-off point was assessed via searching for the maximum value of sensitivity + specificity–1 (Youden index). Area under curve (AUC) was calculated to evaluate the predictive powers. All the analyses were performed with SPSS version 19.0 for Windows (SPSS Inc, Chicago, III, USA). P values < 0.05 were defined as statistically significant.

## Results

### Maternal lipid profile by trimester

Table 1 shows maternal lipid profile by trimester. Serum TC, TG, LDL-C, HDL-C concentrations increased as pregnancy advanced. All the lipid parameters of the third trimester were higher than those of the first and second trimesters (*p* < 0.001). Furthermore, the increases in lipid concentrations from the second to third trimester were larger than those from the first to second trimester (*p* < 0.001). From the second to third trimester, TC, TG, LDL-C and HDL-C concentrations increased by 35.5 %, 24.1 %, 13.4 % and 9.0 %, respectively. In contrast, the data was separately 12.9 % (TC), 12.3 % (TG), 10.2 % (LDL-C), 4.8 % (HDL-C) from the first to second trimester.

**Table 1:** Maternal lipid profile by trimester

Lipids	First	Second	Third	First-Second	Second-Third	p*	p**
TC	3.95 (3.66-4.60)	4.65 (4.22-5.10)	6.27 (5.52-7.03)	0.51 (0.21-0.87)	1.65 (1.04-2.29)	0.000	0.000
TG	2.20 (1.77-2.73)	2.45 (2.11-2.89)	3.06 (2.37-3.98)	0.27 (0.09-0.51)	0.59 (0.12-1.11)	0.000	0.000
LDL-C	2.25 (2.08-2.45)	2.46 (2.22-2.77)	2.87 (2.32-3.45)	0.23 (0.03-0.46)	0.33 (−0.09-0.84)	0.000	0.000
HDL-C	1.66 (1.45-1.77)	1.67 (1.47-1.79)	1.80 (1.57-2.04)	0.08 (−0.12-0.21)	0.15 (−0.09-0.32)	0.000	0.000

*TC* total cholesterol, *TG* triglycerides, *LDL-C/HDL-C* low-density/high density lipoprotein-cholesterol

Maternal lipid concentrations and increases were presented as median (IQR) mmol/L

*p values were derived from the comparisons among lipid concentrations of the first, second, and third trimesters

**p value were derived from the comparisons between lipid increases from the first to second trimester and the second to third trimester

### Characteristics of study population

Table 2 presents maternal and neonatal characteristics of our study population. Among the 934 eligible mothers in the present study, the mean (SD) age at delivery was 29.21 (3.76) years old. Approximately 83 % of them were nulliparous and about 7 % of them were stratified as overweight or obese with prepregnancy BMI ≥ 25.0 kg/m^2^. Their mean (SD) prepregnancy BMI was 20.66 (2.70) kg/m^2^. The prevalence of maternal comorbidities, including GDM, preeclampsia and ICP was respectively 7.6 %, 1.5 % and 6.3 %. The mean (SD) gestational weight gain was 15.27 (4.00) kg. According to the IOM recommendations for gestational weight gain, 54.0 % met, 15.4 % fell below and 30.6 % exceeded the criteria. The newborns in our study had a mean (SD) birth weight of 3396.41 (436.52) g. 71.3 % of them were AGA, 2.4 % were SGA and 26.3 % were LGA. 1.3 % of them belonged to low birth weight group and 8.0 % belonged to macrosomia group. The mean (SD) gestational age at birth was 38.84 (1.22) weeks.

**Table 2:** Maternal and neonatal characteristics

Characteristics	Mean ± SD or N (%)
Maternal characteristics	
Maternal age at delivery (year)	29.21 ± 3.76
20-29	569 (60.9)
30-34	285 (30.5)
≥35	80 (8.6)
Prepregnancy BMI (kg/m^2^)	20.66 ± 2.70
Underweight (<18.5)	192 (20.7)
Normal weight (18.5-24.9)	669 (72.1)
Overweight and obese (≥25.0)	67 (7.2)
Gestational weight gain	15.27 ± 4.00
Appropriate	501 (54.0)
Inadequate	143 (15.4)
Excessive	284 (30.6)
Maternal education	
≥University	591 (63.3)
Junior college	296 (31.7)
High school	36 (3.9)
≤Junior school	8 (0.9)
Unknown	3 (0.3)
Family income (yuan/year)	
>200,000	33 (3.5)
100,000-200,000	756 (80.9)
50,000-100,000	118 (12.6)
<50,000	19 (2.0)
Unknown	8 (0.9)
Parity	
Nulliparous	778 (83.3)
Multiparous	156 (16.7)
Exposed to cigarette during pregnancy	59 (6.3)
GDM	71 (7.6)
Preeclampsia	14 (1.5)
ICP	59 (6.3)
Delivery mode	
Vaginal delivery	216 (23.1)
Cesarean section	718 (76.9)
Neonatal characteristics	
Male infant	493 (52.8)
Gestational age (GA) at delivery	38.84 ± 1.22
Preterm (GA < 37 weeks)	27 (2.9)
Full term (37 ≤ GA < 42 weeks)	907 (97.1)
Birth weight(g)	3396.41 ± 436.52
<2500	12 (1.3)
2500-4000	847 (90.7)
>4000	75 (8.0)
Weight for gestational age	
SGA	22 (2.4)
AGA	666 (71.3)
LGA	246 (26.3)

*BMI* body mass index, *GDM* gestational diabetes mellitus, *ICP* intrahepatic cholestasis of pregnancy, *SGA/AGA/LGA* small/appropriate/large for gestational age

### Associations between maternal lipid profile and pregnancy complications and perinatal outcomes

Table 3 displays the associations between maternal third-trimester lipid profile and pregnancy complications and perinatal outcomes. Of all the expectant mothers, 7.6 % developed GDM, 1.5 % preeclampsia and 6.3 % ICP. We observed every mmol/L elevation in maternal third-trimester TG concentration was associated with an increased risk of GDM [*p* < 0.001, adjusted odds ratio (AOR) = 1.37, 95 % confidence interval (CI): 1.18-1.58], preeclampsia (*p* = 0.002, AOR = 1.50, 95 % CI: 1.16-1.93) and ICP (*p* = 0.002, AOR = 1.28, 95 % CI: 1.09-1.51). In addition, every unit increase in LDL-C reduced the risk of GDM (*p* < 0.001, AOR = 0.52, 95 % CI: 0.38-0.72). While on the other hand, there were no significant associations between TC concentrations and all the above pregnancy complications.

**Table 3:** Associations between maternal third-trimester lipid profile and pregnancy complications and perinatal outcomes

	TC		TG		LDL-C		HDL-C	
Outcomes	AOR (95%CI)	*p* value	AOR (95%CI)	*p* value	AOR (95%CI)	*p* value	AOR (95%CI)	*p* value
GDM	0.84 (0.68-1.04)	0.109	1.37 (1.18-1.58)	0.000	0.52 (0.38-0.72)	0.000	0.27 (0.13-0.58)	0.001
Preeclampsia	0.99 (0.64-1.54)	0.979	1.50 (1.16-1.93)	0.002	0.65 (0.33-1.31)	0.228	0.48 (0.10-2.26)	0.352
ICP	1.20 (0.97-1.49)	0.096	1.28 (1.09-1.51)	0.002	1.29 (0.94-1.76)	0.113	0.56 (0.25-1.22)	0.141
PTB	0.80 (0.57-1.11)	0.180	1.04 (0.77-1.38)	0.818	0.83 (0.52-1.32)	0.430	1.05 (0.37-3.00)	0.931
SGA	1.12 (0.80-1.56)	0.520	0.63 (0.40-0.99)	0.046	1.16 (0.71-1.89)	0.565	3.15 (1.15-8.65)	0.026
LGA	0.98 (0.86-1.11)	0.715	1.13 (1.02-1.26)	0.025	0.93 (0.78-1.11)	0.418	0.79 (0.52-1.21)	0.281
Macrosomia	0.99 (0.81-1.21)	0.903	1.19 (1.02-1.39)	0.024	0.93 (0.69-1.25)	0.621	0.46 (0.22-0.94)	0.034

*AOR* adjusted odds ratio, *CI* confidence interval, *GDM* gestational diabetes mellitus, *ICP* intrahepatic cholestasis of pregnancy, *PTB* preterm birth, *SGA/LGA* small/large for gestational age, *TC* total cholesterol, *TG* triglycerides, *LDL-C/HDL-C* low-density/high-density lipoprotein-cholesterol

Odds ratios were adjusted for maternal age, prepregnancy BMI, gestational weight gain, parity, maternal education background, family income and cigarette exposure. Values of PTB, SGA, LGA and macrosomia were additionally corrected for delivery mode and infant sex

In our study, the incidence of PTB, SGA, LGA and macrosomia was 2.9 %, 2.4 %, 26.3 % and 8.0 %, respectively. Table 3 showed there was no significant association between third-trimester TG and PTB. However, multivariate analysis discovered that every mmol/L increase in third-trimester TG concentration was associated with an increased risk for LGA (*p* = 0.025, AOR = 1.13, 95 % CI: 1.02-1.26) and macrosomia (*p* = 0.024, AOR = 1.19, 95 % CI: 1.02-1.39), a decreased risk for SGA (*p* = 0.046, AOR = 0.63, 95 % CI: 0.40-0.99). In contrast, there were no significant associations between TC and LDL-C concentrations and all the above perinatal outcomes.

Table 4 reports the associations between maternal HDL-C concentrations and GDM, macrosomia and SGA at all gestational stages. Significant negative associations were observed between HDL-C and the risk for GDM (during the whole pregnancy) and macrosomia (during the second and third trimesters). The strongest inverse associations appeared during the second trimester. Every unit increase in second-trimester HDL-C concentration was associated with a decreased risk for GDM (*p* < 0.001, AOR = 0.10, 95 % CI: 0.03-0.31) and macrosomia (*p* = 0.011, AOR = 0.25, 95 % CI: 0.09-0.73). Additionally, women with higher HDL-C concentrations were at a greater risk of having SGA offsprings and the relationship reached statistical significance at the third trimester (*p* = 0.026, AOR = 3.15, 95 % CI: 1.15-8.65).

**Table 4:** Associations between maternal HDL-C and GDM, macrosomia and SGA

	GDM		Macrosomia		SGA	
Trimester	AOR (95%CI)	*p* value	AOR (95%CI)	*p* value	AOR (95%CI)	*p* value
First	0.15 (0.05-0.42)	0.000	0.51 (0.19-1.36)	0.178	1.31 (0.32-5.38)	0.709
Second	0.10 (0.03-0.31)	0.000	0.25 (0.09-0.73)	0.011	1.88 (0.47-7.59)	0.377
Third	0.27 (0.13-0.58)	0.001	0.46 (0.22-0.94)	0.034	3.15 (1.15-8.65)	0.026

*AOR* adjusted odds ratio, *CI* confidence interval, *GDM* gestational diabetes mellitus, *SGA* small for gestational age, *HDL-C* high-density lipoprotein-cholesterol

Odds ratios were adjusted for maternal age, prepregnancy BMI, gestational weight gain, parity, maternal education background, family income and cigarette exposure. Values of macrosomia and SGA were additionally corrected for delivery mode and infant sex

Table 5 presents the optimal cut-off points of maternal third-trimester lipids for predicting pregnancy complications and perinatal outcomes. The optimal cut-off points proposed by ROC curve analysis for third-trimester TG in predicting GDM, preeclampsia, ICP, LGA and SGA were separately ≥3.871, 3.528, 3.177, 3.534 and ≤2.530 mmol/L. TG predicting preeclampsia owned the strongest predictive power with the largest AUC [0.736 (95 % CI: 0.595-0.876)]. The optimal cut-off points for third-trimester HDL-C in identifying GDM, macrosomia and SGA were respectively ≤1.712, 1.817 and ≥2.238 mmol/L. HDL-C identifying GDM had the strongest predictive ability with the largest AUC [0.617 (95 % CI: 0.548-0.686)]. Besides, the optimal cut-off point for third-trimester LDL-C in predicting GDM was ≤2.415 mmol/L.

**Table 5:** Optimal cut-off points of maternal third-trimester lipids for predicting pregnancy complications and perinatal outcomes

Outcomes	AUC (95 % CI)	Sensitivity (%)	Specificity (%)	Youden index	Cut-off point (mmol/L)
TG					
GDM	0.633 (0.566-0.701)	47.9	74.2	0.221	3.871
Preeclampsia	0.736 (0.595-0.876)	85.7	64.8	0.505	3.528
ICP	0.607 (0.528-0.685)	66.1	55.9	0.220	3.177
LGA	0.571 (0.529-0.613)	44.7	67.4	0.121	3.534
SGA	0.658 (0.540-0.775)	63.6	68.9	0.325	2.530
HDL-C					
GDM	0.617 (0.548-0.686)	57.7	61.2	0.189	1.712
Macrosomia	0.592 (0.527-0.657)	66.7	49.8	0.165	1.817
SGA	0.606 (0.457-0.756)	40.9	88.7	0.296	2.238
LDL-C					
GDM	0.649 (0.582-0.715)	54.9	71.7	0.266	2.415

*AUC* area under curve, *CI* confidence interval, *GDM* gestational diabetes mellitus, *ICP* intrahepatic cholestasis of pregnancy, *SGA/LGA* small/large for gestational age, *TG* triglycerides, *HDL-C/LDL-C* high-density/low-density lipoprotein-cholesterol

Except for the last trimester, per unit elevation in TG concentrations could led to 1.58-fold increased risk of GDM in the first trimester (*p* = 0.003, AOR = 1.58, 95 % CI: 1.17-2.15) and 1.55-fold increased risk in the second trimester (*p* = 0.001, AOR = 1.55, 95 % CI: 1.19-2.02). Moreover, high TG at the second trimester were significantly associated with the morbidity of ICP (*p* = 0.006, AOR = 1.50, 95 % CI: 1.12-2.00). Pregnant women with higher LDL-C during the second trimester were at reduced risk of GDM (*p* = 0.010, AOR = 0.48, 95 % CI: 0.28-0.84).

## Discussion

In this population-based study, we comprehensively displayed maternal fasting lipid profile by trimester in Chinese reproductive-age women and explored the associations between maternal lipid concentrations and pregnancy complications and perinatal outcomes. The present study confirmed maternal TG concentrations in pregnancy were positively associated with the risk of GDM, preeclampsia, LGA and macrosomia among Chinese population, which was in line with previous research performed in other races/ethnicities [14–20, 24, 31]. Furthermore, we found maternal high HDL-C concentrations were significantly associated with a decreased risk for GDM and macrosomia, and an increased risk for SGA. At the same time, hypertriglyceridemia during pregnancy was a significant predictor of ICP. These were the main novel discoveries in our study, suggesting complex metabolic disorders of maternal lipids were potentially important factors that involved in the pathological processes of adverse pregnancy outcomes.

One striking finding from this study was that maternal HDL-C concentrations were negatively associated with the risk for GDM and macrosomia, and positively associated with the risk for SGA. These associations indicated maternal HDL-C concentrations beyond a certain range was an underlying predictor of GDM, macrosomia or SGA. Very few relevant reports could be found in the past [34, 47, 48]. Among them, Wang et al. [47] believed insulin resistance, relative lack of insulin secretion and oxidative stress were the main contributors of maternal hypertriglyceridemia and low HDL-C concentrations, especially in GDM pregnancies.

Another interesting finding was that maternal high TG concentrations during the second and third trimesters were associated with an increased risk of ICP. This phenomenon has rarely been reported in the prior studies as far as we know. Evidence from a recent prospective study suggested that ICP was related with higher TG concentrations and lower HDL-C concentrations under fasting conditions [49]. Currently, theoretical explanations provided by Fiorucci et al. [50] and Sharma et al. [51] implied reduction in the activity of bile acid receptors FXR and TGR5 may be responsible for the above conclusion, however, the potential mechanisms still deserve further exploration.

Our study advanced the understandings of previous controversies and helped to elucidate these issues. One dispute was concerned with LDL-C concentrations in GDM pregnant women. In past studies, certain researchers observed a tendency of lower LDL-C concentrations in expectant mothers with GDM and some of them even discovered atherogenic alterations in LDL particles (increased small size, dense LDL particles and raised LDL susceptibility to oxidation) [16, 35, 38]. But there were contradictory reports stating no significant difference of LDL-C concentrations between diabetic and nondiabetic pregnancies [34, 37]. For this argument, our results supported an inverse association between LDL-C concentrations and the prevalence odds of GDM, especially during the second and third trimesters. In our opinion, the conflicting consequences implied maternal lipid concentrations were influenced by complicated factors (e.g. condition of glycemic control, trimester of pregnancy and race/ethnicity).

There exists another debate focused on the correlations between maternal TG concentrations and neonatal body measurements. It has been demonstrated in some publications that maternal TG concentrations were positively correlated with neonatal birth weight or fat mass in pregnancies complicated by GDM or diabetes [28–30, 33, 39]; whereas in other articles TG concentrations were positively related with fetal development in subjects with normal glucose tolerance [31, 32]. Interestingly, many scholars once hypothesized that excessive fetal growth is derived from GDM or GIGT themselves. That means the aforementioned positive correlation may be interfered by maternal glycemic conditions. Recently, more and more studies verified the hypothesis, suggesting GDM was an independent risk factor for macrosomia [52, 53]. Consequently, our results emphasized maternal TG concentrations were independently and positively correlated with neonatal birth weight, in accordance with the viewpoints of Amsterdam Born Children and their Development cohort study [24, 54].

Compared with the existing findings, there was an inconsistent outcome in our study. In contrast with previous studies [24, 25], no significant associations were observed between maternal TG concentrations and the occurrence of PTB according to our results, whatever the gestational stage was. From our perspective, the conflicting outcomes may be explained by racial/ethnical differences, which means fetuses in different ethnic groups are disproportionally affected by maternal lipid metabolism. Future parallel studies are required to explore the underlying mechanisms of this discrepancy.

Our study has several attractive strengths. It provided some prospective evidence suggesting that dislipidemia during pregnancy places Chinese expectant mothers and their offspring at greater risk of adverse outcomes. Although lipid metabolism in pregnancy has been explored, there are controversies regarding its associations with pregnancy complications and fetal development. To our knowledge, few studies have investigated the relationship in Asian countries, particularly in China. Compared with the study conducted by Wang et al. [47] which assessed the associations between TG/HDL-C ratios and the risk of GDM and LGA, we separately identified the contributions of TG and HDL-C to pregnancy outcomes and expanded insights into effects of different lipid components on mothers and neonates. Indeed, a majority of existing trials focused on the concentrations of TG, TC, LDL-C and their influences on maternal and perinatal outcomes, however, less attention had been paid to HDL-C. Our study not only described the variation trend of HDL-C concentrations by trimester, but also evaluated its role in developing GDM, macrosomia and SGA. Moreover, we proposed the optimal cut-off points of maternal third-trimester lipids in predicting adverse pregnancy outcomes, which was a new contribution has rarely appeared in previous research.

However, the present study still has some limitations. First of all, our sample lacked population diversity. Given that China is a country characterized by huge population and imbalanced regional economic development, a multicenter study with larger sample size can be more representative. Secondly, we selected Neonatal Birth Weight for Gestational Age and Percentile in 15 Cities of China as the criteria for LGA and SGA infants in our analysis. Defect of this criteria is its applicability worths further discussion with the increasing Chinese neonatal birth weights. Thirdly, some collected data of maternal characteristics (e.g. prepregnancy weight and GWG) were self-reported and possibly be subject to recall bias. Finally, we cannot rule out the possibility of residual confounding. For example, excessive dietary nutrition intake and inadequate physical activity during pregnancy may be important contributors of dyslipidemia, but we failed to include sufficient information on dietary nutrition intake and physical activity. Thus, additional studies are needed to clarify the relationship between maternal lifestyle and dyslipidemia and pregnancy outcomes.

## Conclusions

Our results suggested that maternal high TG concentrations in late pregnancy were independently and significantly associated with an increased risk of GDM, preeclampsia, ICP, LGA, macrosomia and a decreased risk of SGA among Chinese population. Additionally, relative low HDL-C during pregnancy was significantly related with the occurence of GDM and macrosomia; whereas relative high HDL-C was a protective factor for both of them. Our findings highlighted the importance of maternal lipid metabolism in preventing pregnancy complications and adverse birth outcomes.

## Abbreviations

AGA: Appropriate for gestational age
AOR: Adjusted odds ratio
AUC: Area under curve
BMI: Body mass index
CVD: Cardiovascular diseases
DOHaD: Developmental origins of Health and Disease
GDM: Gestational diabetes mellitus
GIGT: Gestational impaired glucose tolerance
GWG: Gestational weight gain
HDL-C: High-density lipoprotein-cholesterol
ICP: Intrahepatic cholestasis of pregnancy
IQR: Interquartile range
LDL-C: Low-density lipoprotein-cholesterol
LGA: Large for gestational age
NEFA: Non-esterified fatty acids
OGTT: Oral glucose tolerance test
PTB: Preterm birth
SD: Standard deviation
SGA: Small for gestational age
TC: Total cholesterol
TG: Triglycerides

## References

[1] Kaaja RJ, Greer IA (2005). Manifestations of chronic disease during pregnancy. JAMA.

[2] Lekva T, Bollerslev J, Norwitz ER, Aukrust P, Henriksen T, Ueland T (2015). Aortic Stiffness and Cardiovascular Risk in Women with Previous Gestational Diabetes Mellitus. PLoS One.

[3] Zoet GA, Koster MP, Velthuis BK, de Groot CJ, Maas AH, Fauser BC (2015). Determinants of future cardiovascular health in women with a history of preeclampsia. Maturitas.

[4] Charlton F, Tooher J, Rye KA, Hennessy A (2014). Cardiovascular risk, lipids and pregnancy: preeclampsia and the risk of later life cardiovascular disease. Heart Lung Circ.

[5] Yang JJ, Lee SA, Choi JY, Song M, Han S, Yoon HS (2015). Subsequent risk of metabolic syndrome in women with a history of preeclampsia: data from the Health Examinees Study. J Epidemiol.

[6] Gillman MW (2005). Developmental origins of health and disease. N Engl J Med.

[7] Prentice AM, Moore SE (2005). Early programming of adult diseases in resource poor countries. Arch Dis Child.

[8] Bartels Ä, Egan N, Broadhurst DI, Khashan AS, Joyce C, Stapleton M (2012). Maternal serum cholesterol levels are elevated from the 1st trimester of pregnancy: A cross-sectional study. J Obstet Gynaecol.

[9] Brizzi P, Tonolo G, Esposito F, Puddu L, Dessole S, Maioli M, Milia S (1999). Lipoprotein metabolism during normal pregnancy. Am J Obstet Gynecol.

[10] Piechota W, Staszewski A (1992). Reference ranges of lipids and apolipoproteins in pregnancy. Eur J Obstet Gynecol Reprod Biol.

[11] Lippi G, Albiero A, Montagnana M, Salvagno GL, Scevarolli S, Franchi M, Guidi GC (2007). Lipid and lipoprotein profile in physiological pregnancy. Clin Lab.

[12] Husain F, Latif S, Uddin M, Nessa A (2008). Lipid profile changes in second trimester of pregnancy. Mymensingh Med J.

[13] Ghio A, Bertolotto A, Resi V, Volpe L, Di Cianni G (2011). Triglyceride metabolism in pregnancy. Adv Clin Chem..

[14] Hollingsworth DR, Grundy SM (1982). Pregnancy-associated hypertriglyceridemia in normal and diabetic women. Differences in insulin-dependent, non-insulin-dependent, and gestational diabetes. Diabetes.

[15] Koukkou E, Watts GF, Lowy C (1996). Serum lipid, lipoprotein and apolipoprotein changes in gestational diabetes mellitus: a cross-sectional and prospective study. J Clin Pathol.

[16] Sánchez-Vera I, Bonet B, Viana M, Quintanar A, Martín MD, Blanco P (2007). Changes in plasma lipids and increased low-density lipoprotein susceptibility to oxidation in pregnancies complicated by gestational diabetes: consequences of obesity. Metabolism.

[17] Wiznitzer A, Mayer A, Novack V, Sheiner E, Gilutz H, Malhotra A, Novack L. Association of lipid levels during gestation with preeclampsia and gestational diabetes mellitus: a population-based study. Am J Obstet Gynecol. 2009;201(5):482.e1-8. 10.1016/j.ajog.2009.05.032 PMC5483324 19631920

[18] Hubel CA, McLaughlin MK, Evans RW, Hauth BA, Sims CJ, Roberts JM (1996). Fasting serum triglycerides, free fatty acids, and malondialdehyde are increased in preeclampsia, are positively correlated, and decrease within 48 hours post partum. Am J Obstet Gynecol.

[19] Baker AM, Klein RL, Moss KL, Haeri S, Boggess K. Maternal serum dyslipidemia occurs early in pregnancy in women with mild but not severe preeclampsia. Am J Obstet Gynecol. 2009;201(3):293.e1-4. 10.1016/j.ajog.2009.05.037 19631926

[20] Spracklen CN, Smith CJ, Saftlas AF, Robinson JG, Ryckman KK (2014). Maternal hyperlipidemia and the risk of preeclampsia: a meta-analysis. Am J Epidemiol.

[21] Gratacós E, Casals E, Gómez O, Llurba E, Mercader I, Cararach V, Cabero L (2003). Increased susceptibility to low density lipoprotein oxidation in women with a history of pre-eclampsia. BJOG.

[22] Sanchez SE, Williams MA, Muy-Rivera M, Qiu C, Vadachkoria S, Bazul V (2005). A case–control study of oxidized low density lipoproteins and preeclampsia risk. Gynecol Endocrinol.

[23] Qiu C, Phung TT, Vadachkoria S, Muy-Rivera M, Sanchez SE, Williams MA (2006). Oxidized low-density lipoprotein (oxidized LDL) and the risk of preeclampsia. Physiol Res.

[24] Vrijkotte TG, Krukziener N, Hutten BA, Vollebregt KC, van Eijsden M, Twickler MB (2012). Maternal lipid profile during early pregnancy and pregnancy complications and outcomes: the ABCD study. J Clin Endocrinol Metab.

[25] Catov JM, Bodnar LM, Kip KE, Hubel C, Ness RB, Harger G, Roberts JM. Early pregnancy lipid concentrations and spontaneous preterm birth. Am J Obstet Gynecol. 2007;197(6):610.e1-7. 10.1016/j.ajog.2007.04.024 18060950

[26] Herrera E (2002). Lipid metabolism in pregnancy and its consequences in the fetus and newborn. Endocrine.

[27] Herrera E, Ortega-Senovilla H (2014). Lipid metabolism during pregnancy and its implications for fetal growth. Curr Pharm Biotechnol.

[28] Herrera E, Ortega-Senovilla H (2010). Disturbances in lipid metabolism in diabetic pregnancy - Are these the cause of the problem?. Best Pract Res Clin Endocrinol Metab.

[29] Schaefer-Graf UM, Graf K, Kulbacka I, Kjos SL, Dudenhausen J, Vetter K, Herrera E (2008). Maternal lipids as strong determinants of fetal environment and growth in pregnancies with gestational diabetes mellitus. Diabetes Care.

[30] Ortega-Senovilla H, Schaefer-Graf U, Meitzner K, Abou-Dakn M, Herrera E (2013). Decreased concentrations of the lipoprotein lipase inhibitor angiopoietin-like protein 4 and increased serum triacylglycerol are associated with increased neonatal fat mass in pregnant women with gestational diabetes mellitus. J Clin Endocrinol Metab.

[31] Kitajima M, Oka S, Yasuhi I, Fukuda M, Rii Y, Ishimaru T (2001). Maternal serum triglyceride at 24–32 weeks' gestation and newborn weight in nondiabetic women with positive diabetic screens. Obstet Gynecol.

[32] Di Cianni G, Miccoli R, Volpe L, Lencioni C, Ghio A, Giovannitti MG (2005). Maternal triglyceride levels and newborn weight in pregnant women with normal glucose tolerance. Diabet Med.

[33] Göbl CS, Handisurya A, Klein K, Bozkurt L, Luger A, Bancher-Todesca D, Kautzky-Willer A (2010). Changes in serum lipid levels during pregnancy in type 1 and type 2 diabetic subjects. Diabetes Care.

[34] Ryckman KK, Spracklen CN, Smith CJ, Robinson JG, Saftlas AF (2015). Maternal lipid levels during pregnancy and gestational diabetes: a systematic review and meta-analysis. BJOG.

[35] Qiu C, Rudra C, Austin MA, Williams MA (2007). Association of gestational diabetes mellitus and low-density lipoprotein (LDL) particle size. Physiol Res.

[36] Savona-Ventura C, Vassallo J, Craus J, Anastasiou E, Jotic A, Lalic NM, et al. Biological and biochemical characteristics of a Mediterranean population with Gestational Diabetes Mellitus. J Perinat Med. 2015. doi: 10.1515/jpm-2015-0027. 10.1515/jpm-2015-0027 26021548

[37] Retnakaran R, Qi Y, Connelly PW, Sermer M, Hanley AJ, Zinman B (2010). The graded relationship between glucose tolerance status in pregnancy and postpartum levels of low-density lipoprotein cholesterol and apolipoprotein B in young women: implications for future cardiovascular risk. J Clin Endocrinol Metab.

[38] Rizzo M, Berneis K, Altinova AE, Toruner FB, Akturk M, Ayvaz G (2008). Atherogenic lipoprotein phenotype and LDL size and subclasses in women with gestational diabetes. Diabet Med.

[39] Nolan CJ, Riley SF, Sheedy MT, Walstab JE, Beischer NA (1995). Maternal serum triglyceride, glucose tolerance and neonatal birth weight ratio in pregnancy. Diabetes Care.

[40] World Health Organization. Obesity: preventing and managing the global epidemic. Report of a WHO Consultation. World Health Organ Tech Rep Ser. 2000; 894:i-xii,1-253. 11234459

[41] Rasmussen KM, Yaktine AL, Institute of Medicine (US) and National Research Council (US) Committee to Reexamine IOM Pregnancy Weight Guidelines (2009). Weight Gain During Pregnancy: Reexamining the Guidelines.

[42] Metzger BE, Gabbe SG, International Association of Diabetes and Pregnancy Study Groups Consensus Panel (2010). International association of diabetes and pregnancy study groups recommendations on the diagnosis and classification of hyperglycemia in pregnancy. Diabetes Care.

[43] Wei Y, Yang H, Zhu W, Yang H, Li H, Yan J, Zhang C (2014). International Association of Diabetes and Pregnancy Study Group criteria is suitable for gestational diabetes mellitus diagnosis: further evidence from China. Chin Med J (Engl).

[44] Eiland E, Nzerue C, Faulkner M (2012). Preeclampsia 2012. J Pregnancy..

[45] Williamson C, Geenes V (2014). Intrahepatic cholestasis of pregnancy. Obstet Gynecol.

[46] Scientific research collaborative group of neonatal development in 15 cities of China. Neonatal birth weight for gestational age and percentile in 15 cities of China. Zhonghua Er Ke Za Zhi (Chinese). 1989;27:316.

[47] Wang D, Xu S, Chen H, Zhong L, Wang Z (2015). The associations between triglyceride to high-density lipoprotein cholesterol ratios and the risks of gestational diabetes mellitus and large-for-gestational-age infant. Clin Endocrinol (Oxf).

[48] Li G, Kong L, Zhang L, Fan L, Su Y, Rose JC, Zhang W (2015). Early pregnancy maternal lipid profiles and the risk of gestational diabetes mellitus stratified for body mass index. Reprod Sci.

[49] Martineau MG, Raker C, Dixon PH, Chambers J, Machirori M, King NM (2015). The metabolic profile of intrahepatic cholestasis of pregnancy is associated with impaired glucose tolerance, dyslipidemia, and increased fetal growth. Diabetes Care.

[50] Fiorucci S, Mencarelli A, Palladino G, Cipriani S (2009). Bile-acid-activated receptors: targeting TGR5 and farnesoid-X-receptor in lipid and glucose disorders. Trends Pharmacol Sci.

[51] Sharma R, Long A, Gilmer JF (2011). Advances in bile acid medicinal chemistry. Curr Med Chem.

[52] Wahabi HA, Fayed AA, Alzeidan RA, Mandil AA (2014). The independent effects of maternal obesity and gestational diabetes on the pregnancy outcomes. BMC Endocr Disord..

[53] He XJ, Qin FY, Hu CL, Zhu M, Tian CQ, Li L (2015). Is gestational diabetes mellitus an independent risk factor for macrosomia: a meta-analysis?. Arch Gynecol Obstet.

[54] Vrijkotte TG, Algera SJ, Brouwer IA, van Eijsden M, Twickler MB (2011). Maternal triglyceride levels during early pregnancy are associated with birth weight and postnatal growth. J Pediatr.

